# Patterns of Genetic And Epigenetic Diversity Across A Range Expansion in The White-Footed Mouse (*Peromyscus Leucopus*)

**DOI:** 10.1093/iob/obad038

**Published:** 2023-10-30

**Authors:** T L Rubi, J R do Prado, L L Knowles, B Dantzer

**Affiliations:** Department of Psychology, University of Michigan, Ann Arbor, MI, USA; Departamento de Ciências Biológicas, Escola Superior de Agricultura ‘Luiz de Queiroz’, Universidade de São Paulo, Piracicaba, SP, Brazil; Department of Ecology and Evolutionary Biology, University of Michigan, Ann Arbor, MI, USA; Department of Psychology, University of Michigan, Ann Arbor, MI, USA; Department of Ecology and Evolutionary Biology, University of Michigan, Ann Arbor, MI, USA

## Abstract

Populations at the leading front of a range expansion must rapidly adapt to novel conditions. Increased epigenetic diversity has been hypothesized to facilitate adaptation and population persistence via non-genetic phenotypic variation, especially if there is reduced genetic diversity when populations expand (i.e., epigenetic diversity compensates for low genetic diversity). In this study, we use the spatial distribution of genetic and epigenetic diversity to test this hypothesis in populations of the white-footed mouse (*Peromyscus leucopus*) sampled across a purported recent range expansion gradient. We found mixed support for the epigenetic compensation hypothesis and a lack of support for expectations for expansion populations of mice at the range edge, which likely reflects a complex history of expansion in white-footed mice in the Upper Peninsula of Michigan. Specifically, epigenetic diversity was not increased in the population at the purported edge of the range expansion in comparison to the other expansion populations. However, input from an additional ancestral source populations may have increased genetic diversity at this range edge population, counteracting the expected genetic consequences of expansion, as well as reducing the benefit of increased epigenetic diversity at the range edge. Future work will expand the focal populations to include expansion areas with a single founding lineage to test for the robustness of a general trend that supports the hypothesized compensation of reduced genetic diversity by epigenetic variation observed in the expansion population that was founded from a single historical source.

## Introduction

Populations at the leading front of a range expansion or invasion must rapidly adapt to novel conditions to successfully colonize new environments. Often, such populations also lack genetic variation due to low population size, bottleneck effects, founder effects, limited gene flow, and assortative mating (Excoffier et al. 2009; Phillips et al. 2010), theoretically compromising their ability to persist in these new environments (Excoffier et al. 2009; Phillips et al. 2010). Phenotypic plasticity, where environmentally-induced phenotypic variation arises from non-genetic (i.e., epigenetic) effects, may provide an important source of phenotypic variation and promote population persistence in novel conditions (Chevin and Lande 2010; Chevin et al. 2010) or range expansions (Lande 2015). (Though we note that plasticity can be in part genetically determined, and plasticity itself can respond to selection; Ashander et al. 2016; Chevin and Hoffman 2017; Kingsolver and Buckley 2017; Scheiner et al. 2017). Phenotypic plasticity in the absence of genetic variation can produce novel phenotypes very rapidly, even within a single generation. As such, epigenetically induced plasticity has been hypothesized to act as an adaptive buffer for populations experiencing environmental change by facilitating persistence until evolutionary (genetic) adaptation can occur (Price et al. 2003; Excoffier et al. 2009; O'Dea et al. 2016).

Recent theoretical and empirical evidence supports the hypothesis that phenotypic plasticity due to epigenetic effects, in particular deoxyribonucleic acid (DNA) methylation, can facilitate colonization of novel habitats and ecological range expansions (reviewed in Hawes et al. 2018). Most work to date has focused on the relationship between genetic diversity and epigenetic diversity, here defined narrowly as variation in single methylation polymorphisms (SMPs), analogous to SNP variation (e.g., Liebl et al. 2013, Schmitz et al. 2013). A growing body of empirical work shows that epigenetic diversity is often increased relative to genetic diversity in colonist populations. For example, invasive populations show high levels of epigenetic diversity and low levels of genetic diversity relative to native populations in Japanese knotweed (*Fallopia spp.*; Richards et al. 2012), annual bluegrass (*Poa annua*; Chwedorzewska and Bednarek 2012), and the carpet sea squirt (*Didemnum vexillum*; Hawes et al. 2019). In repeated introductions, this compensatory relationship may be greatest in the most recently established populations. For example, populations of house sparrows (*Passer domesticus*) from a recent invasion (<50 years ago) compared to an older invasion (150 years ago) had reduced genetic diversity, but epigenetic diversity was similar in the two populations (Schrey et al. 2012; see also Sheldon et al. 2018). In other words, epigenetic diversity was elevated relative to genetic diversity in younger colonist populations compared to older colonist populations, consistent with the hypothesis that epigenetic diversity can compensate for reduced genetic diversity. Similar trends were also observed in the pygmy mussel (*Xenostrobus secures*) and the Australian tubeworm (*Ficopomatus enigmacticus*; Ardura et al. 2017). These effects are expected to be stronger in invasive populations that experienced pronounced population bottlenecks, however, populations at the front of range expansions may experience similar effects due to repeated bottlenecks, assortative mating, and low population densities (Excoffier et al. 2009; Phillips et al. 2010, Canestrelli et al. 2016). Less work has been done measuring trends across range expansions. Liebl et al. (2013) found evidence for epigenetic compensation across a range expansion of house sparrows (Passer domesticus) in Kenya, though they did not find a clear spatial trend across the expansion gradient. Taken together, these studies suggest that epigenetic diversity may compensate for reduced genetic diversity by providing a rapid, non-genetic source of phenotypic variation that could promote the persistence of colonist populations.

**Fig. 1:**
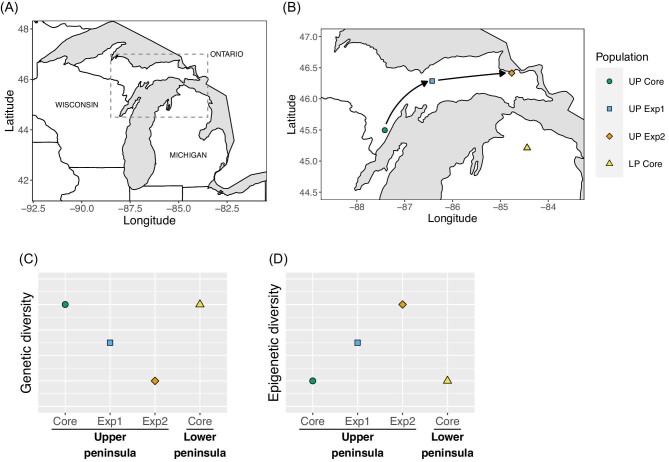
Range expansion region and predicted results. (A) Great Lakes region of North America. The region shown in (B) is indicated by the dashed box. (C) Sampled populations in northern Michigan: UP Core (Menominee county), UP Exp1 (Schoolcraft county), UP Exp2 (Chippewa county), and LP Core (Cheboygan county). The arrows indicate the predicted single-source range expansion model, with mice moving northeastward from source populations in the western UP. (C) Genetic diversity predictions. If increased epigenetic diversity compensates for reduced genetic diversity at the expansion front, genetic diversity will be greatest in the core populations and decrease across the northeastward expansion gradient in the UP. (D) Epigenetic diversity predictions. Epigenetic diversity, in contrast, will be lowest in the core populations and increase across the northeastward expansion gradient in the UP.

In this study, we measure epigenetic and genetic diversity across an ecological range expansion of the white-footed mouse (*Peromyscus leucopus noveboracensis*). We then test whether spatial patterns in diversity are consistent with the hypothesis that increased epigenetic diversity compensates for reduced genetic diversity at the leading edge of the expansion. We characterize epigenetic variation using DNA methylation, an epigenetic marker associated with transcriptional regulation and modulation of gene expression. *Peromyscus leucopus* is a native North American rodent that is currently shifting its range northward, likely due to shorter and milder winter conditions (Long 1996; Myers et al. 2005, 2009; Garcia-Elfring et al. 2017). It is the most common mammal species within its range, and is broadly distributed across forests in the northeastern United States and into southeastern Canada. We focus here on the Upper Peninsula (UP) of Michigan (Fig. 1**A**) where regular small mammal censuses in the region indicated that *P. leucopus* was absent in the UP prior to 1981 except in the southernmost county of the UP (Menominee), where they were documented in natural history collections that began in 1939 (Myers et al. 2009). Since 1981, *P. leucopus* has been trapped with increasing frequency and is now found in most regions of the UP (Myers et al. 2009). Trapping records also suggest a northeastward expansion of a single lineage across the UP at an estimated rate of 15 km/year (Fig. 1**B**; Myers et al. 2009) with populations in the eastern UP considered to be the most recently established. Morphological analyses also suggest mice in these different populations have adjusted to local environmental conditions using plasticity (Baumgartner and Hoffman 2019).

Using this background, we sampled white-footed mice collected from four sites in northern Michigan to test the prediction that epigenetic (methylation) diversity compensates for reduced genetic diversity in expansion populations (Fig. 1). These samples were collected as part of a broader project investigating both spatial and temporal patterns in genetic and epigenetic diversity using historic museum specimens (see Rubi et al. 2020, or Card et al. 2021 and Raxworthy and Smith 2021 for more information on the challenges and benefits of using museum specimens for genomic analysis). For this reason, we sampled tissue from dried skulls, one of the most common preparations found in historical museum collections. We used a combination of double digest restriction site-associated DNA sequencing (ddRAD) and bisulfite treatment to generate genome-wide reduced representation genomes and methylomes to estimate genetic diversity and epigenetic diversity in each population. We predicted that genetic diversity would be greatest in the core populations and decrease along the expansion gradient (see Fig. 1**C**), while epigenetic diversity would be lowest in the core populations and increase along the expansion gradient (see Fig. 1**D**).

In a companion analysis conducted concurrently with the work presented here (Prado et al. 2022), we used the same raw dataset to test multiple hypotheses of geographic range expansion in the UP. In contrast to the expansion model presented above, Prado et al. (2022) found evidence for an older, postglacial colonization of the UP from two different ancestral sources, an eastern source and a western source. Below we describe the trends in genetic and epigenetic diversity measured in this study and interpret these trends based on the previous expansion model as well as the updated model proposed by Prado et al. (2022).

## Materials and methods

### Geographic sampling and specimens

We sampled three sites in the UP and one in the Lower Peninsula (LP) of Michigan, which were categorized as either expansion populations (i.e., populations that were established after 1981) or core populations (populations from the historic range). The two core populations occur at a similar latitude and represent the estimated northern range limit of the species up to 1981 (based upon trapping/census records: Myers et al. 2009). Hereafter, we refer to these populations as the UP core population (UP Core; Menominee county) and the LP core population (LP Core; Cheboygan county) (Fig. 1**B**). We also sampled two expansion populations within the UP. The first, UP expansion population 1 (UP Exp1; Schoolcraft county), is located approximately 120 km northeastward of UP Core and represents the estimated range edge in 2002. The second, UP expansion population 2 (UP Exp2; Chippewa county), is located 230 km northeastward of UP Core and represents the range edge in 2016. Data on sample collection, including the site and date of sampling, are shown in Supplementary Table S1.

We sampled tissue from 62 adult white-footed mouse specimens (*P. leucopus*) vouchered at the University of Michigan Museum of Zoology or Miami University (specimen numbers and details are shown in Supplementary Table S1) following guidelines of the American Society of Mammalogists (Sikes 2016). White-footed mice are sympatric with some other *Peromyscus* species (especially *Peromsycus maniculatus gracilis*) in some of our study areas, but species identity can be discerned visually (Underhill et al. 2021). All specimens were collected from 2013 to 2016 between July and October. When possible, sampling was balanced between the sexes. (see Supplementary Table S1). All permits from the United States Forest Service or State of Michigan were obtained by the field collection teams for these museum vouchered specimens.

### Tissue sampling and DNA extraction

Because DNA methylation is highly tissue-specific, it is necessary to standardize the tissue sampled across individuals. We sampled bone tissue from traditionally prepared dried museum skulls. To minimize damage to the skulls, we sampled microturbinates (small nasal bones) by inserting a sterile micropick into the nasal cavity to dislodge 5–12 mg of tissue (Wisely et al. 2004; Taylor and Hoffman 2010). Prior to DNA extraction, the bone fragments were placed into thick-walled 2 mL microcentrifuge tubes with four 2.4 mm stainless steel beads and processed in a FastPrep tissue homogenizer (MP Biomedicals) for 1 min at 6.0 m/s. DNA was extracted using the Qiagen DNeasy Blood and Tissue Kit, with modifications for working with museum specimens (see Rubi et al. 2020 for full extraction protocol). All pre-amplification steps were performed under a laminar flow hood in the Ancient DNA Laboratory at the University of Michigan, a dedicated laboratory for processing low quality specimens, and followed stringent anti-contamination protocols (unidirectional flow of equipment and personnel, filtered pipette tips, additional negative controls, etc.).

### Library preparation

All 62 samples were sequenced in both a regular ddRAD library to measure genetic variation and a bisulfite treated ddRAD library to measure cytosine methylation variation (as described in Rubi et al. 2020). The libraries were prepared together at the beginning of the protocol and then split in half prior to bisulfite treatment. We digested each sample with the restriction enzymes SphI-HF and MluCI for 1 h at 37°C (New England Biolabs). These enzymes were chosen because they are insensitive to DNA methylation (and therefore will not show biased template enrichment) and have previously been used to prepare libraries in *Peromyscus* (Munshi-South et al. 2016). We added a spike-in of digested unmethylated lambda phage DNA (Sigma Aldrich) to each sample at a concentration of 0.1% of the sample concentration; these phage reads were used to directly measure the bisulfite conversion rate for each individual sample. Samples were individually barcoded using custom methylated Illumina adapters (Sigma Aldrich), pooled, and size-selected for fragments ranging from 376 to 412 bp using a Pippin Prep electrophoresis platform (Sage Biosciences). The size-selected libraries were then split in half. One half was amplified for regular genetic sequencing, and the other half underwent bisulfite conversion to determine cytosine methylation. Bisulfite conversion was performed using a Promega MethylEdge Bisulfite Conversion Kit, which converted unmethylated cytosines to uracils, and the converted DNA was amplified by polymerase chain reaction using KAPA HiFi HotStart Uracil + MasterMix, which replaced uracils with thymines in the amplified product. The library was sequenced in one lane for 100 bp paired-end reads on an Illumina HiSeq 2500 (San Diego, CA, USA).

### Genetic data processing

We processed raw sequence reads in STACKS v.2.41 (Catchen et al. 2013). We checked reads for adapter contamination, demultiplexed, and filtered using the *process_radtags* script allowing one mismatch in the adapter sequence and a barcode distance of one (–adapter_mm 1 –barcode_dist 1). We trimmed all reads to 96 bp, and only reads with Phred score >10 and unambiguous barcodes were retained. We excluded individuals with fewer than 200,000 raw reads, as low-quality specimens can result in assembly problems and excessive filtering of loci.

We aligned the resulting paired-end reads for each individual to the *P. leucopus* genome (NCBI assembly ID: GCA_004664715.1) using Bowtie2 (Langmead and Salzberg 2012). We performed read mapping using default parameters (maximum and minimum mismatch penalties were 6 and 2, respectively). Some read pairs did not overlap in the alignment, increasing the amount of missing data in the final concatenated sequences. As a result, we focus only on forward reads for the genetic analysis. We used a read depth minimum of 3X.

We extracted the consensus sequence for each assembled locus using *gstacks* with default options and processed these files in *populations* to identify loci across populations (–p 2). We exported the SNP data in Variant Call Format (vcf) and processed in R v. 3.3.2 using the PEGAS package (R Core Team 2017; Paradis 2010). We removed SNPs from the last 16 base pairs of all loci due to an increased frequency of polymorphisms, as well as loci with high theta values (both are suggestive of sequencing and assembly errors). We ran all STACKS modules under parallel execution with eight threads in the University of Michigan ARC cluster.

### Epigenetic data processing

We restricted our analysis to methylation occurring in the context of CpG dinucleotides; in mammals, DNA methylation occurs almost exclusively in this context (Jones and Takai 2001). (In our dataset, around 1% of non-CpG cytosines were methylated, versus around 65% of CpG cytosines.) We processed bisulfite sequences as described in Rubi et al. (2020). Briefly, we demultiplexed the raw reads using the *process_radtags* script of STACKS with default settings except a maximum barcode distance of one (–barcode_dist 1) and disabled restriction site check (–disable_rad_check). We trimmed demultiplexed reads for quality and adapter contamination and removed cut site sequences using *TrimGalore* v.0.6.0 (www.bioinformatics.babraham.ac.uk/projects/trim_galore/). After quality processing and adapter trimming, we visually assessed the reads for degradation at read ends using Mbias plots. We removed cut site sequences by trimming 5 positions from the 5ʹ end of forward reads (–clip_r1 5) and 4 positions from the 5ʹ end of reverse reads (–clip_r2 4). To remove low quality positions, we further trimmed forward reads by 3 positions at the 5ʹ end and truncated reads to 119 bp at the 3ʹ end (–hardtrim5 118).

We aligned paired-end reads to the *P. leucopus* genome and performed methylation calling using the bisulfite aligner *Bismark* v.0.18.1 (Krueger and Andrews 2011) with default settings except for the mismatch criteria (–N 1) and gap penalties (–score_min L,0,-0.4), which allowed for more differences between the aligned reads and the reference. We used *Bowtie2* v.2.1.0 (Langmead and Salzberg 2012) as the core aligner. We also aligned the reads to the lambda phage genome (NCBI ID: NC_001416) using default alignment settings and used these reads to estimate the bisulfite conversion rate for each sample.

We further filtered the methylation calls output by Bismark based on the sample-specific bisulfite conversion rate using functions from *MethylExtract* v.1.9 (Barturen et al. 2013). Briefly, we used Bismark to generate a list of all CpG positions in our sequences with the number of methylated and unmethylated reads. We then estimated the sample-specific bisulfite conversion rates from the lambda phage-aligned reads using the *MethylExtractBSCR* function. We determined significant methylation calls using the *MethylExtractBSPvalue* function, which assigns *P*-values to each CpG based on binomial tests incorporating the raw read counts and the sample-specific bisulfite conversion rate and uses the Benjamini–Hochberg step-up procedure to control the false discovery rate for multiple testing. We specified an FDR of 0.2. Only significant sites with a minimum read depth of 5X were used in downstream analyses.

We used this dataset to calculate the % methylation at one randomly selected CpG dinucleotide per amplicon (analyzing a random site is one method to deal with spatial correlation in the methylation levels of neighboring CpG sites). Although CpG methylation shows tissue-specificity, it is highly variable and even within a single cell type the methylation state of a given CpG position in the genome may vary between individual cells. As a result, methylation at a given position is typically expressed as a percentage ranging from fully methylated (methylated in 100% of reads) to fully unmethylated (methylated in 0% of reads). Usually, a given CpG position sampled within a single tissue type is either fully methylated or fully unmethylated. Less commonly, methylation of a CpG position varies between cells; such positions are called partially methylated (e.g., methylated in 80% of reads; Rakyan et al. 2004; Eckhardt et al. 2006).

### Sample dropout and supplementary analyses

We calculated genetic and epigenetic diversity statistics for all loci present in at least three individuals in all four populations. After all filtering steps, the final dataset for diversity analyses included only 39 individuals: 17 from LP Core, 6 from UP Core, 4 from UP Exp1, and 12 from UP Exp2. This high level of dropout is not unusual for museum specimens, which tend to show high variance in the yield of recoverable DNA; however, such low sample sizes have the potential to bias the calculation of diversity statistics. To confirm that our results were not an artifact of low sample size, we conducted two supplementary analyses. First, we subsampled three individuals from all populations and repeated our genetic diversity calculations. This reanalysis yielded the same results as our original analysis, suggesting that sample size did not bias our results. This work is described in our companion paper using the same dataset, Prado et al. 2022 (see Supplementary Data SD4). Second, we used principal component analysis (PCA) (PC1–PC3) to visualize genetic variation in the populations and confirmed that no individuals from the populations with low sample size (UP Exp1, UP Core) were displaced from the rest of the population, as would be expected if a single outlier individual were biasing the statistics (Supplementary Fig. S1). This PCA did recover an outlier individual in the population with the highest sample size, LP Core (MZ11379al; Supplementary Fig. S1). A reanalysis excluding this individual yielded similar diversity statistics (Supplementary Table S2), so we retained this specimen in the final analysis.

### Characterizing genetic and epigenetic diversity

We estimated genetic diversity using observed heterozygosity (*H*_obs_) and nucleotide diversity (π) from a dataset of 5937 loci. We calculated genetic diversity measures using the STACKS module *populations* for loci with a maximum of 25% missing data (selected using PLINK 1.07; Purcell et al. 2007). We estimated epigenetic diversity from 682 SMPs using two measures: the standard deviation of % methylation and unbiased haplotype diversity (uh). To calculate the standard deviation of % methylation, we first calculated % methylation over all reads at each epilocus. We then calculated the standard deviation in % methylation across all epiloci. We performed these analyses using custom scripts in R. We calculated unbiased haplotype diversity using GENALEX v6.5 (Peakall and Smouse 2006). We scored each locus as a methylated epiallele (over 50% of reads methylated) or an unmethylated epiallele (under 50% of reads methylated). These epialleles were input into GENALEX and analyzed as haplotypes.

We compared the mean locus diversity values among populations for all four diversity measures (genetic: *H*_obs_ and π, epigenetic: uh and standard deviation (SD) in % methylation) using non-parametric Kruskal Wallis tests. We conducted post hoc comparisons using Tukey's Honestly Significant Difference tests.

We compared pairwise genetic and epigenetic distances with a Mantel's test using the function mantel from R package VEGAN version 2.5–6 (Oksanen et al. 2013). We also performed a partial mantel correlation analysis between genetic variation, epigenetic variation, and geography using the function “mantel.partial” from the R package VEGAN version 2.5–6 (Oksanen et al. 2013).

## Results

**Fig. 2:**
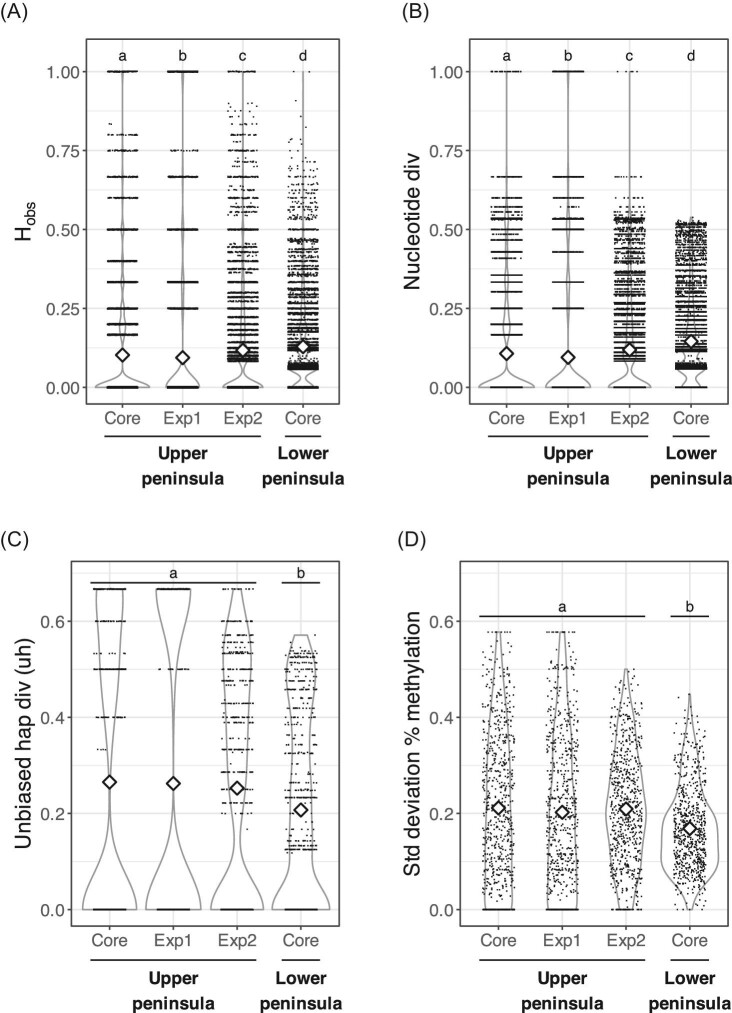
Violin plots of two measures of genetic diversity (A and B) and two measures of epigenetic diversity (C and D) per locus across the mouse populations. Raw data points are overlaid and the means are indicated by the white diamonds, with statistically significant differences shown at the top (indicated by different letters).

As expected, the two genetic diversity measures, observed heterozygosity (*H*_obs_) and nucleotide diversity (π), are highly correlated (*r* = 0.90, *P* < 0.001). For both genetic diversity measures, mean diversity differs significantly across populations, and all pairwise comparisons are significant (*H*_obs_: Kruskal Wallis, χ^2^_3 _= 3434.2, *P* < 0.001; π: Kruskal–Wallis, χ^2^_3 _= 3455.8, *P* < 0.001: Fig. 2**A, B**). Genetic diversity is highest in the LP Core population (*H*_obs _= 0.129+/−0.136; π = 0.146+/−0.136), consistent with our prediction that genetic diversity would be highest in historic populations with larger population sizes, and is higher than in either expansion population: UP Exp1 (*H*_obs _= 0.094+/−0.230; π=0.095+/−0.215), and UP Exp2 (*H*_obs _= 0.117+/−0.179; π=0.119+/−0.157). The genetic diversity of the UP Core population (*H*_obs _= 0.102+/−0.201; π=0.107+/−0.183, Fig. 2**A, B**) is higher than the UP Exp 1, but not the UP Exp2 (Fig. 2**A, B**). The higher genetic diversity of the UP Exp2 population relative to the UP Exp1 population was not what we predicted based on the expansion history informed from field surveys and museum records (Myers et al. 2009). That is, we expected genetic diversity of the core population in the UP (UP Core population in Menominee County) would be higher than the two more recently founded expansion populations (UP Exp1 and UP Exp2), but we only found support for this prediction in one of the populations (UP Exp1).

The two epigenetic diversity measures, unbiased haplotype diversity (uh) and the standard deviation of % methylation (SD), are significantly positively correlated (*r* = 0.80, *P*  < 0.001). Mean epigenetic diversity across loci also differs in both measures, though this difference is only marginally significant for uh when using a p value cutoff of 0.05 (uh: Kruskal–Wallis, χ^2^_3 _= 7.769, *P*  = 0.051; SD: Kruskal–Wallis, χ^2^_3 _= 36.543, *P*  < 0.001, Fig. 2**C, D**). Pairwise comparisons revealed that mean epigenetic diversity does not differ significantly across the three UP populations (UP Core, UP Exp1, UP Exp2: uh = 0.260+/−0.287; SD = 0.208+/−0.146), but epigenetic diversity is significantly lower in the LP Core population (uh = 0.207+/−0.200; SD = 0.169+/−0.087; Fig. 2**C, D**). Low epigenetic diversity in the LP Core population is expected given the corresponding high genetic diversity (Fig. 2**A, B**). However, epigenetic diversity in the UP Core population is similar to the epigenetic diversity in the expansion populations and there is not an increase in epigenetic diversity toward the range edge proposed by Myers et al. (2009) in the UP (i.e., the epigenetic diversity of UP Exp2 was not greater than that of UP Exp1: Fig. 2**C, D**). Unsurprisingly then, genetic diversity and epigenetic diversity are not correlated (pairwise Mantel tests: *r* = −0.6089, *P* = 0.916). A partial Mantel test comparing pairwise genetic and epigenetic distance after controlling for the effect of geographic distance also is not significant (*r* = −0.6523, *P* = 0.875).

## Discussion

The trends in genetic and epigenetic diversity presented here are less consistent with our original expansion model (hereafter the single-source model, e.g., Myers et al. 2009) and more consistent with a range expansion model assuming two different ancestral source populations (hereafter the two-source model, Prado et al. 2022). Most notably, the population at the range edge according to the single-source model (UP Exp 2) shows increased genetic diversity, rather than reduced diversity, relative to the closest putative source population (UP Exp 1) as well as the putative ancestral source (UP Core). Increased genetic diversity in this population is, however, consistent with the two-source model, in which UP Exp 2 represents a contact zone between two separate lineages. The single-source expansion model is supported by trapping data (Myers et al. 2009) and mitochondrial studies (e.g., Rowe et al. 2006). Subsequent analyses using mitochondrial (Moscarella et al. 2019) and microsatellite markers (Baumgartner and Hoffman 2019) identified gene flow from a separate eastern lineage, but proposed rare, recent events such as human-mediated dispersal as the source (see Moscarella et al. 2019 for a discussion). However, a more comprehensive genome-wide analysis indicates divergence times consistent with a longer history of occupancy of the eastern UP by *P. leucopus*, suggesting postglacial colonization of the UP by *both* an eastern and a western lineage (Prado et al. 2022). Although more work needs to be done to confirm these trends, for the purposes of the rest of this paper, we adopt the two-source model.

In light of this updated expansion (two-source) model, a different sampling design will be required to test our hypothesis in this system; however, we can still test updated predictions about diversity and colonization using the data presented here. First, we can test our original predictions for a single-source model by focusing on the westernmost expansion population (UP Exp 1) and its source population (UP Core). In these populations, genetic diversity is significantly higher in the source population and lower in the expansion population, as we expected. Epigenetic diversity, in contrast, does not differ significantly between the two populations. Taken together, these trends support an epigenetic compensation hypothesis because epigenetic variation relative to genetic variation is higher in the colonist population compared to the source population. Similar trends have been found in other colonist systems. Schrey et al. (2012) compared populations of house sparrows (*Passer domesticus*) from a recent invasion (<50 years old) to an older invasion (150 years old) and found that while genetic diversity was greatly reduced in the recent invasion, epigenetic diversity was similar in both populations. Likewise, Ardura et al. (2017) observed the same trends in invasive populations of two marine invertebrate species (pygmy mussel, *X. secures*, and Australian tubeworm, *F. enigmacticus*).

Second, we can make updated predictions about diversity in UP Exp 2, the contact zone between two lineages in the two-source model. Intraspecific admixture of two separate lineages in a colonist population generates novel allelic combinations and counteracts the effects of bottlenecks and inbreeding (see Rius and Darling 2014 for a review), which we in turn expect to reduce the benefit of increased epigenetic diversity. We predict that such populations would more closely resemble non-colonist populations; namely, we expect to see increased genetic diversity and reduced epigenetic diversity. As discussed above, genetic diversity is indeed higher in UP Exp 2 than in the other two UP populations, including the historic source population (UP Core). Epigenetic diversity does not vary significantly between the three UP populations. In other words, epigenetic diversity is lower relative to genetic diversity in this population than in the expansion population (UP Exp 1), consistent with our updated predictions.

It is also worth noting that genetic and epigenetic diversity are not tightly correlated across our sampled populations. The hypothesis that epigenetic variation facilitates colonization by promoting rapid phenotypic plasticity assumes that epigenetic markers (here cytosine methylation) can vary at least partially independently of genetic markers (reviewed in Hawes et al. 2018, Carneiro and Lyko 2020). However, it is not yet fully clear to what extent methylation state varies independently of genetic variation; most known variably methylated cytosines are associated with genetic markers (methyl quantitative trait loci, or meQTL; Taudt et al. 2016). More work is needed describing the relationship between genetic and epigenetic variation, which is of fundamental importance to the compensation hypothesis (Excoffier et al. 2009; Phillips et al. 2010), as well as any hypothesis involving methylation-induced phenotypic plasticity.

Future work can comprehensively test the compensation hypothesis in this system by focusing on western populations in Wisconsin and the western UP, where the expansion appears to be derived from a single, western lineage. Another avenue for future work, whether in this system or others, is to use historic museum specimens to characterize temporal changes in epigenetic and genetic diversity. Measuring diversity in a colonist population over time would be a powerful test of the epigenetic compensation hypothesis. Several studies have successfully characterized cytosine methylation in historic and even ancient tissues, however, obtaining high quality data can be challenging (see Rubi et al. 2020 for work in this system, or Hahn et al. (2020), Niiranen et al. (2022) for recent reviews). Finally, future work could focus on different tissues. Methylation is tissue-specific, and epigenetic diversity might be more variable in certain tissue types. In this study, we chose skull tissue to allow comparisons to historic museum specimens; future work without such constraints might focus on other tissues that are predicted to be “more plastic,” or that are directly related to traits of interest.

## Supplementary Material

obad038_Supplemental_Files

## Data Availability

Data and analysis files are available on FigShare (DOI 10.6084/m9.figshare.24438427). Raw sequence data will be available upon request.
