# Supplementary material for: Patterns of Genetic And Epigenetic Diversity Across A Range Expansion in The White-Footed Mouse (Peromyscus Leucopus)
Source: Integr Org Biol. 2023 Oct 30;5(1):obad038. doi: 10.1093/iob/obad038 (PMC10628966; doi:10.1093/iob/obad038)

Figure S1. Our principal component analysis (PCA) using PC1-PC3 (see main text for description) recovered an outlier individual (MZ11379al) in the lower peninsula ("LP") historical ("Core") population (shown in the lower right hand corner of (a)). We reanalyzed the data without this outlier individual and recovered similar diversity statistics to our original analysis (shown in Table S2). Because the reanalysis did not influence our results and we have no methodological or biological reason to exclude this individual, we retained the individual for our final analysis. Below we show the PCA results for the "All specimens" dataset (a-c) and the "Outlier removed" dataset (d-f). Sampled populations in northern Michigan shown here include UP Core (Menominee county), UP Exp1 (Schoolcraft county), UP Exp2 (Chippewa county), and LP Core (Cheboygan county). **(a)** PCA with all specimens, PC1 and PC2. The outlier specimen (MZ11379al) is the specimen from LP Core shown in the lower right hand corner of the plot. **(b)** PCA with all specimens, PC1 and PC3. **(c)** PCA with all specimens, PC2 and PC3. **(d)** PCA with the outlier specimen removed, PC1 and PC2. **(e)** PCA with the outlier specimen removed, PC1 and PC3. **(f)** PCA with the outlier specimen removed, PC2 and PC3.

(a) PCA All specimens – PC1 and PC2

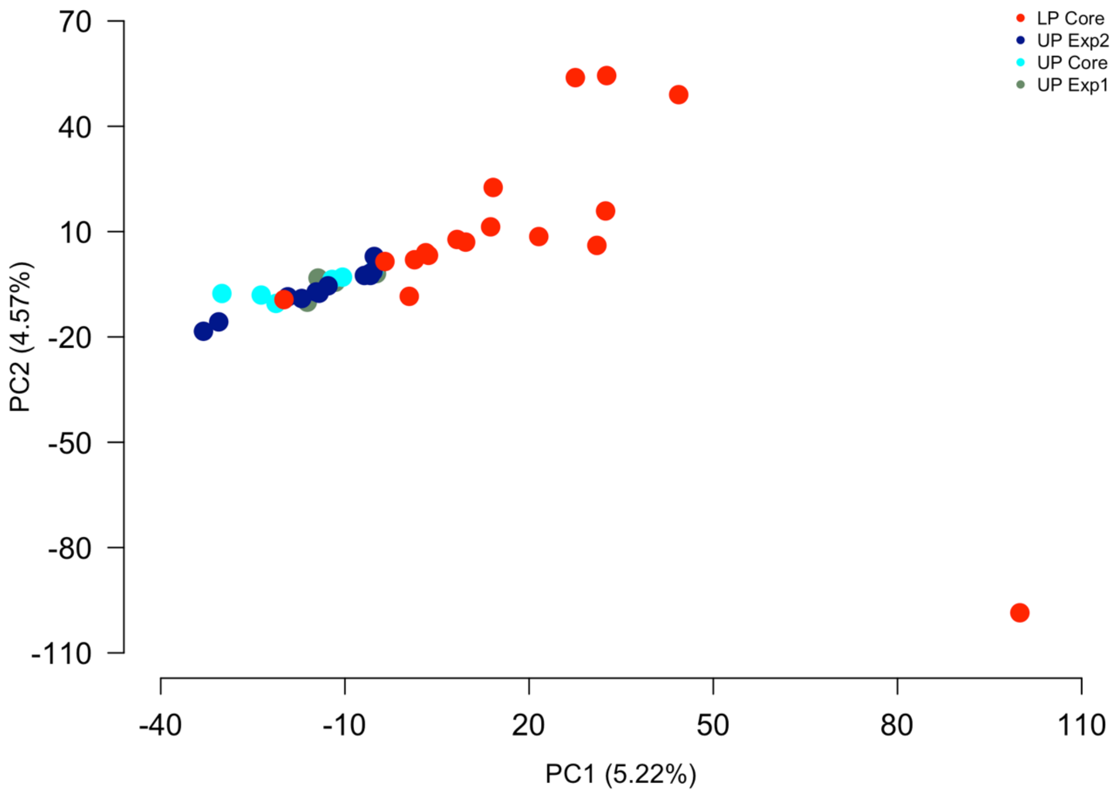

(b) PCA All specimens – PC1 and PC3

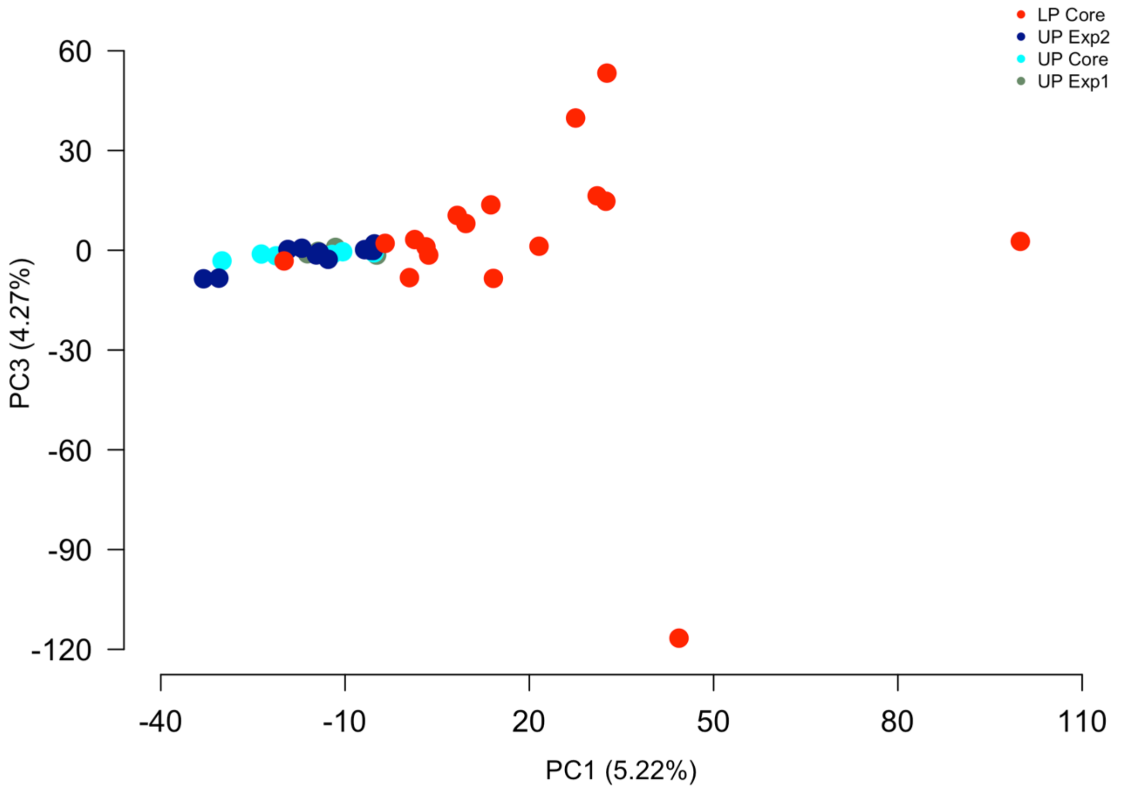

(c) PCA All specimens – PC2 and PC3

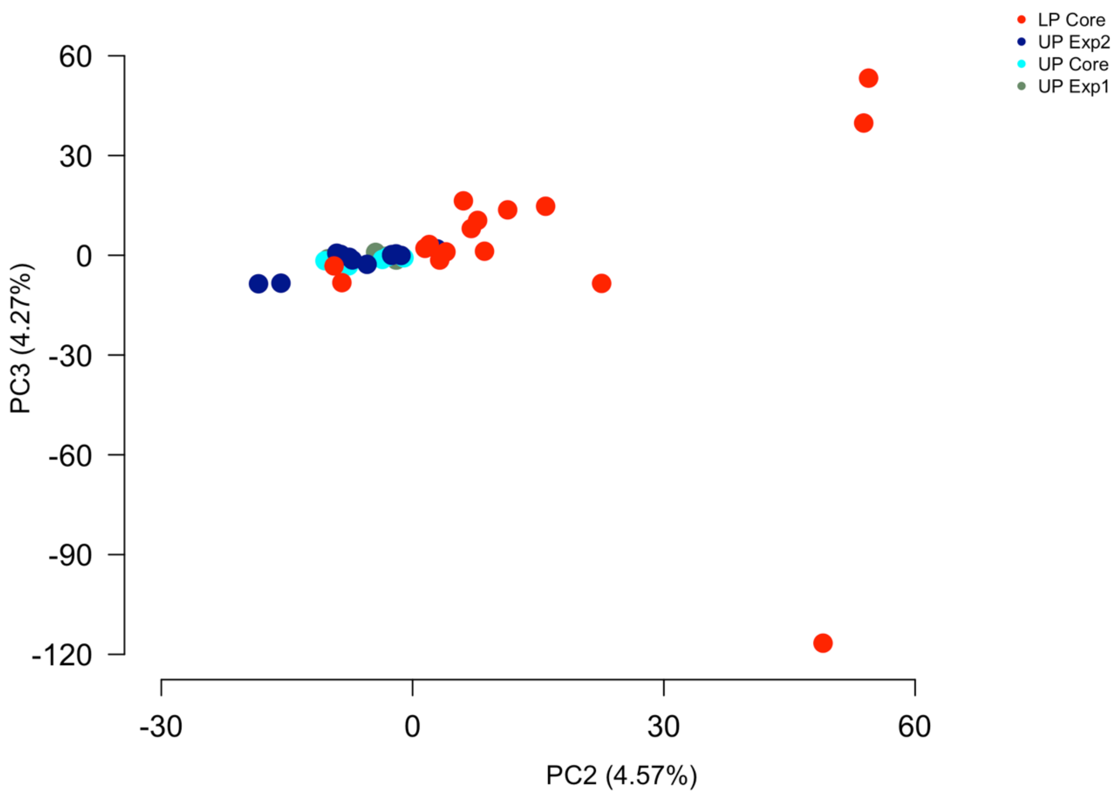

(d) PCA Outlier removed – PC1 and PC2

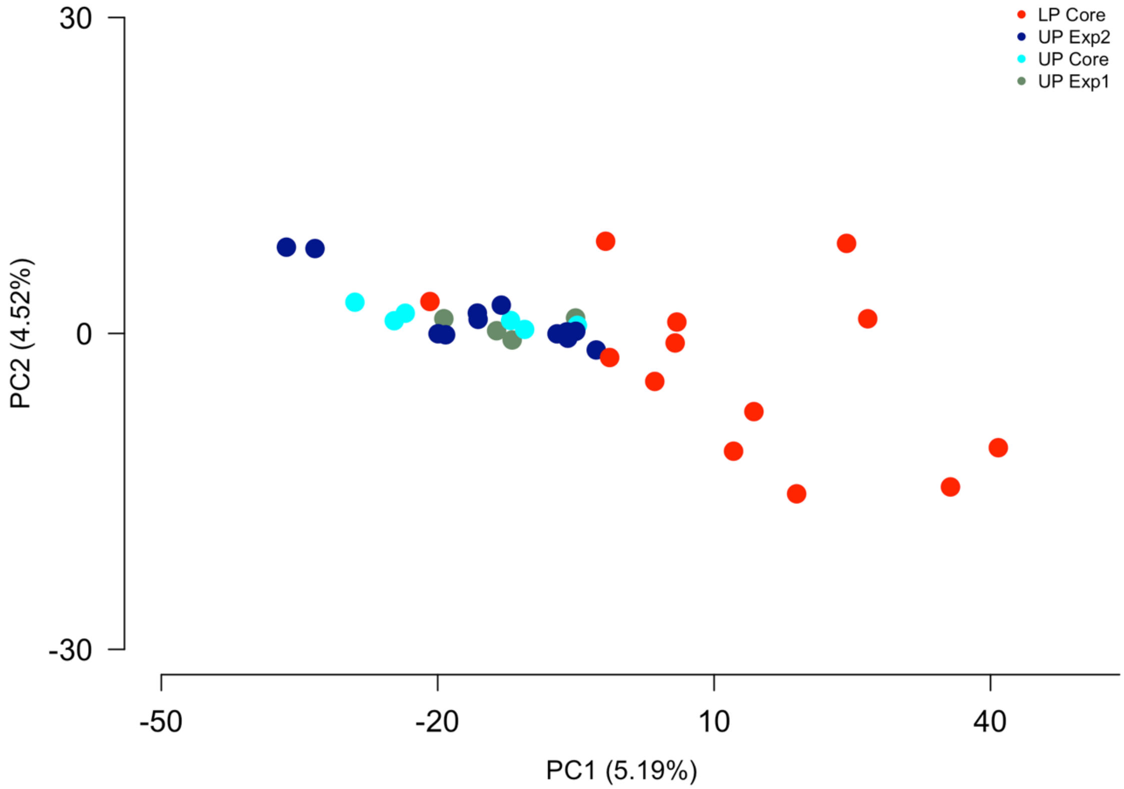

(e) PCA Outlier removed – PC1 and PC3

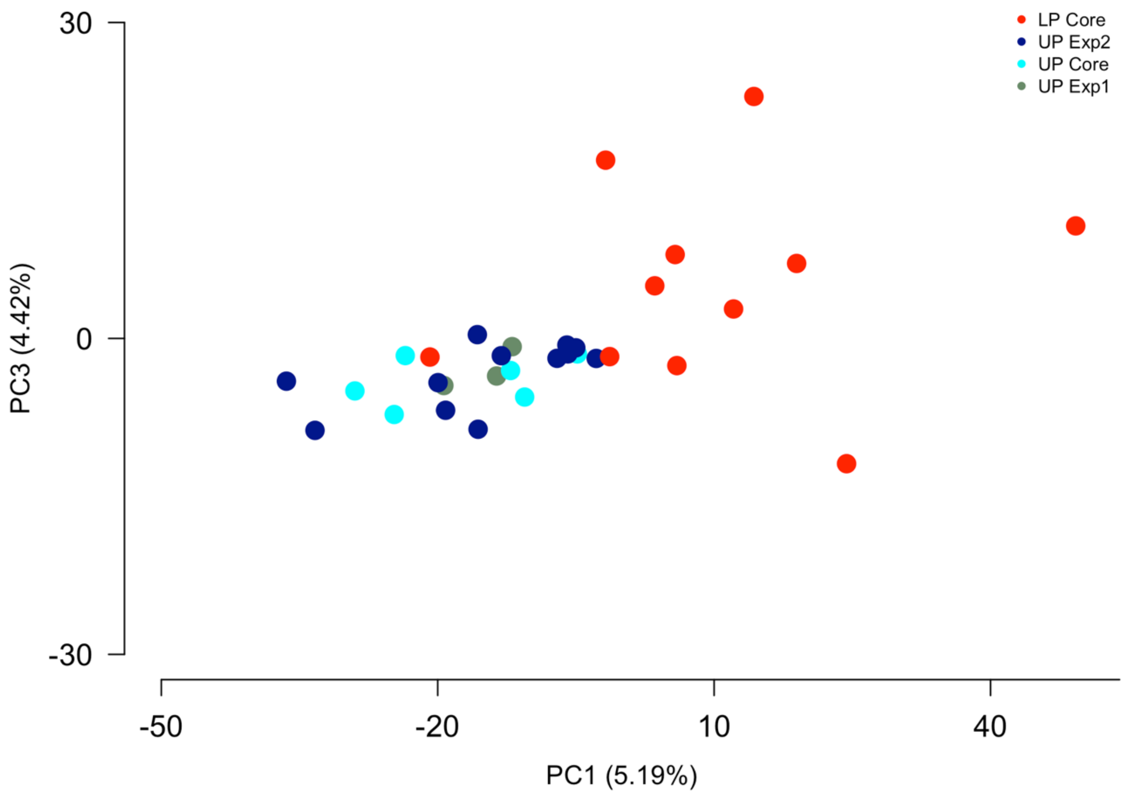

(f) PCA Outlier removed – PC2 and PC3

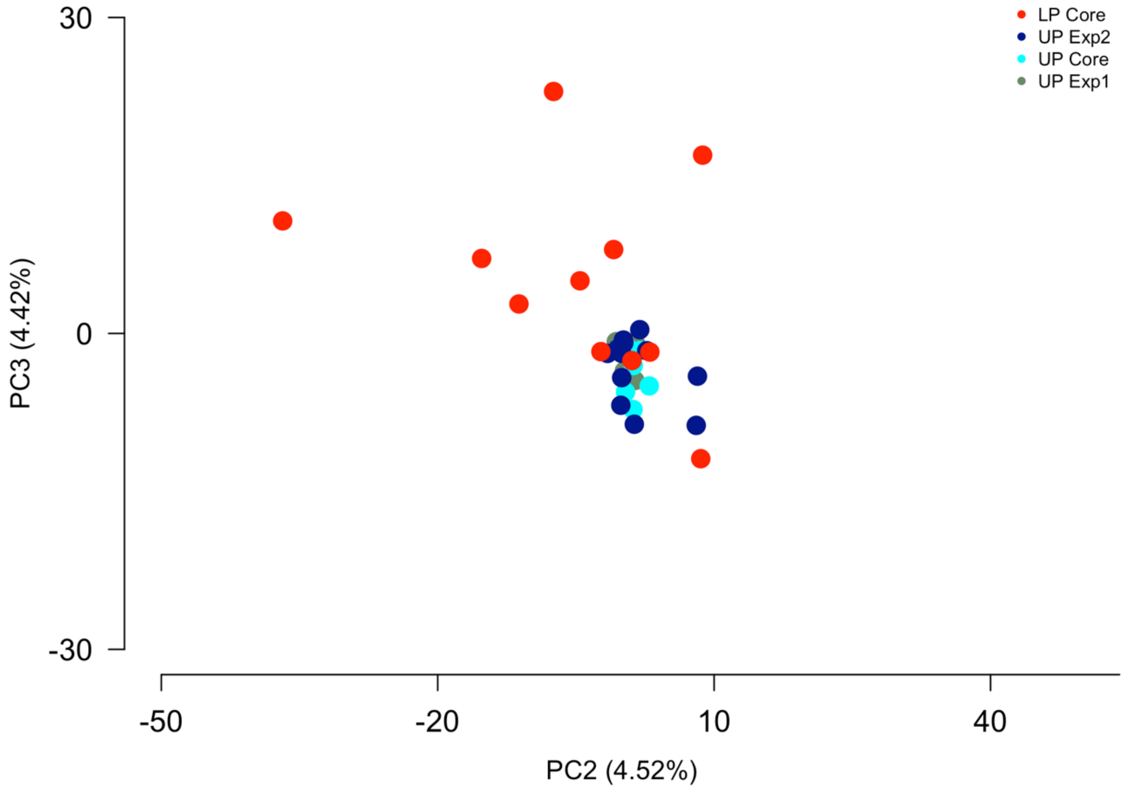

Supplement: obad038_Supplemental_Files [file obad038_supplemental_files.zip › Figure_S1.pdf]
