# Supplementary material for: Patterns of Genetic And Epigenetic Diversity Across A Range Expansion in The White-Footed Mouse (Peromyscus Leucopus)
Source: Integr Org Biol. 2023 Oct 30;5(1):obad038. doi: 10.1093/iob/obad038 (PMC10628966; doi:10.1093/iob/obad038)
Supplement: obad038_Supplemental_Files [file obad038_supplemental_files.zip › supplementary materials first look.pdf]

Title: Patterns of genetic and epigenetic diversity across a range expansion in the white-footed mouse (*Peromyscus leucopus*)

Authors, Affiliations, and ORCID

Tricia L. Rubi, Department of Psychology, University of Michigan, Ann Arbor, MI, USA, tricia.rubi@gmail.com, ORCID: 0000-0003-1333-8898

Joyce R. do Prado, Departamento de Ciências Biológicas, Escola Superior de Agricultura 'Luiz de Queiroz', Universidade de São Paulo, Piracicaba, SP, Brazil, joyce.prado@usp.br, ORCID: 0000-0002-2025-5479

L. Lacey Knowles, Department of Ecology and Evolutionary Biology, University of Michigan, Ann Arbor, MI, USA, knowlesl@umich.edu, ORCID: 0000-0002-6567-4853

Ben Dantzer, Department of Psychology, Department of Ecology and Evolutionary Biology, University of Michigan, Ann Arbor, MI, USA, dantzer@umich.edu, ORCID: 0000-0002-3058-265X

Figure S1. Our principal component analysis (PCA) using PC1-PC3 (see main text for description) recovered an outlier individual (MZ11379al) in the lower peninsula ("LP") historical ("Core") population (shown in the lower right hand corner of (a)). We reanalyzed the data without this outlier individual and recovered similar diversity statistics to our original analysis (shown in Table S2). Because the reanalysis did not influence our results and we have no methodological or biological reason to exclude this individual, we retained the individual for our final analysis. Below we show the PCA results for the "All specimens" dataset (a-c) and the "Outlier removed" dataset (d-f). Sampled populations in northern Michigan shown here include UP Core (Menominee county), UP Exp1 (Schoolcraft county), UP Exp2 (Chippewa county), and LP Core (Cheboygan county). **(a)** PCA with all specimens, PC1 and PC2. The outlier specimen (MZ11379al) is the specimen from LP Core shown in the lower right hand corner of the plot. **(b)** PCA with all specimens, PC1 and PC3. **(c)** PCA with all specimens, PC2 and PC3. **(d)** PCA with the outlier specimen removed, PC1 and PC2. **(e)** PCA with the outlier specimen removed, PC1 and PC3. **(f)** PCA with the outlier specimen removed, PC2 and PC3.

(a) PCA All specimens – PC1 and PC2

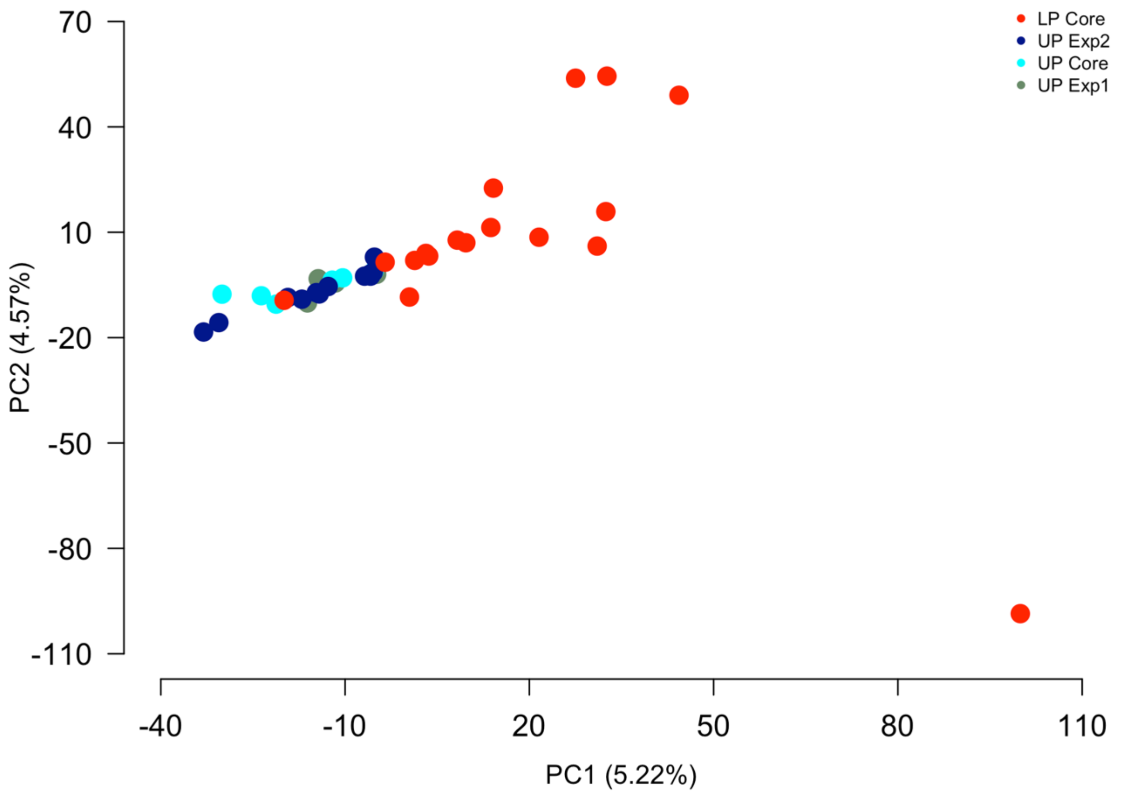

(b) PCA All specimens – PC1 and PC3

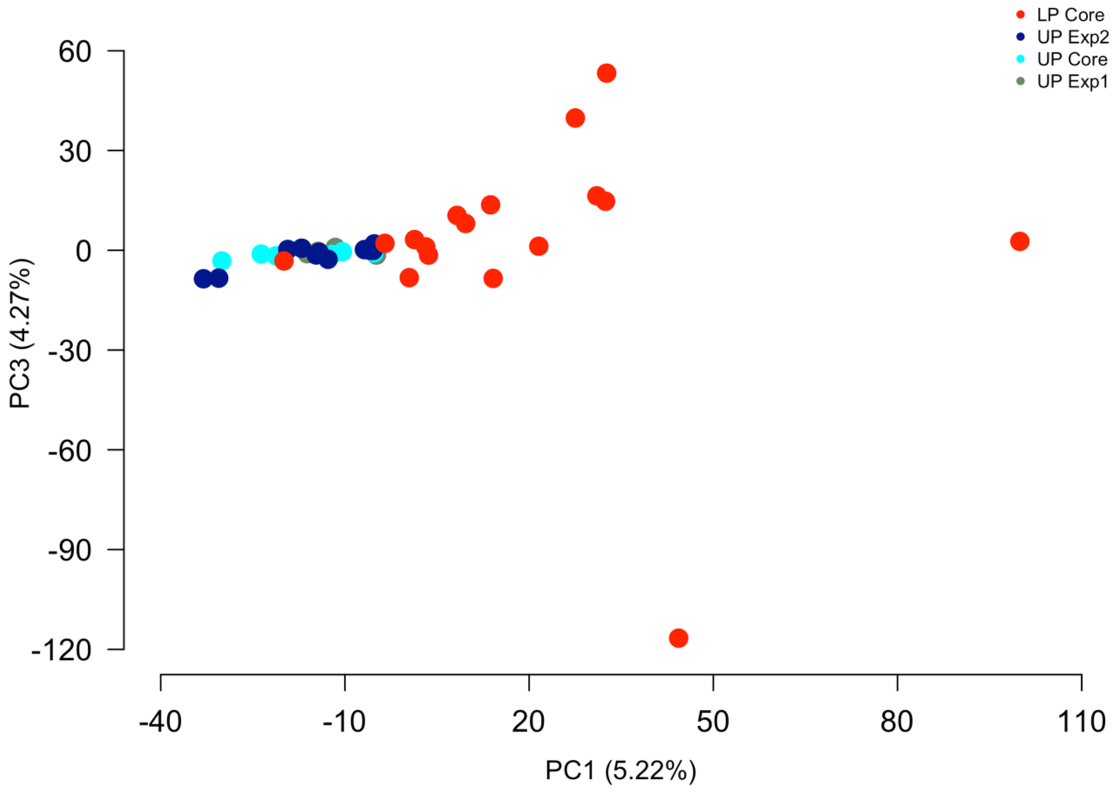

(c) PCA All specimens – PC2 and PC3

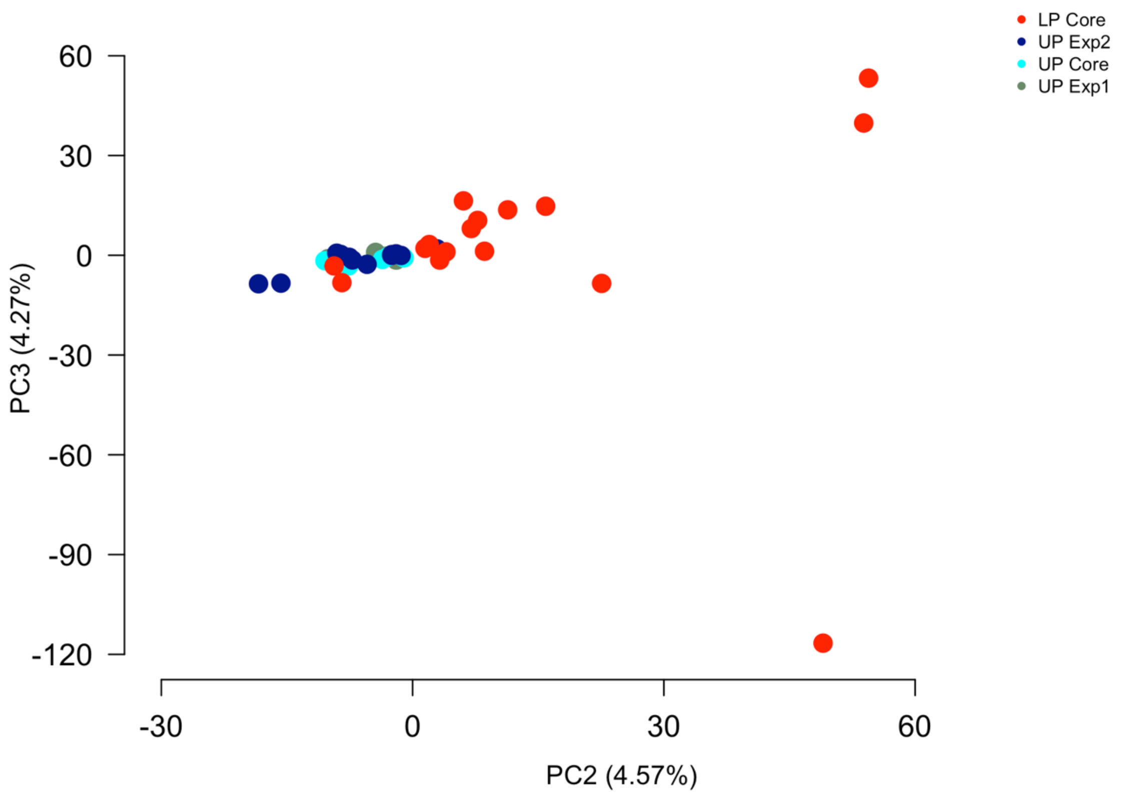

(d) PCA Outlier removed – PC1 and PC2

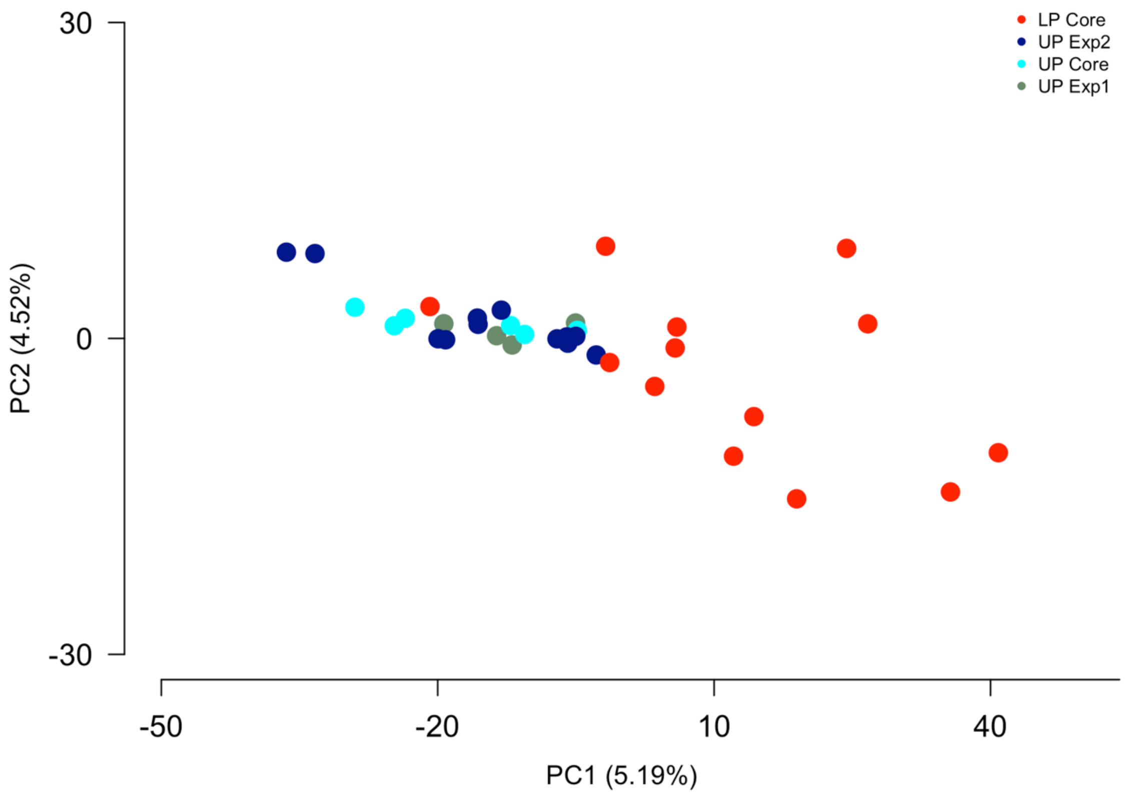

(e) PCA Outlier removed – PC1 and PC3

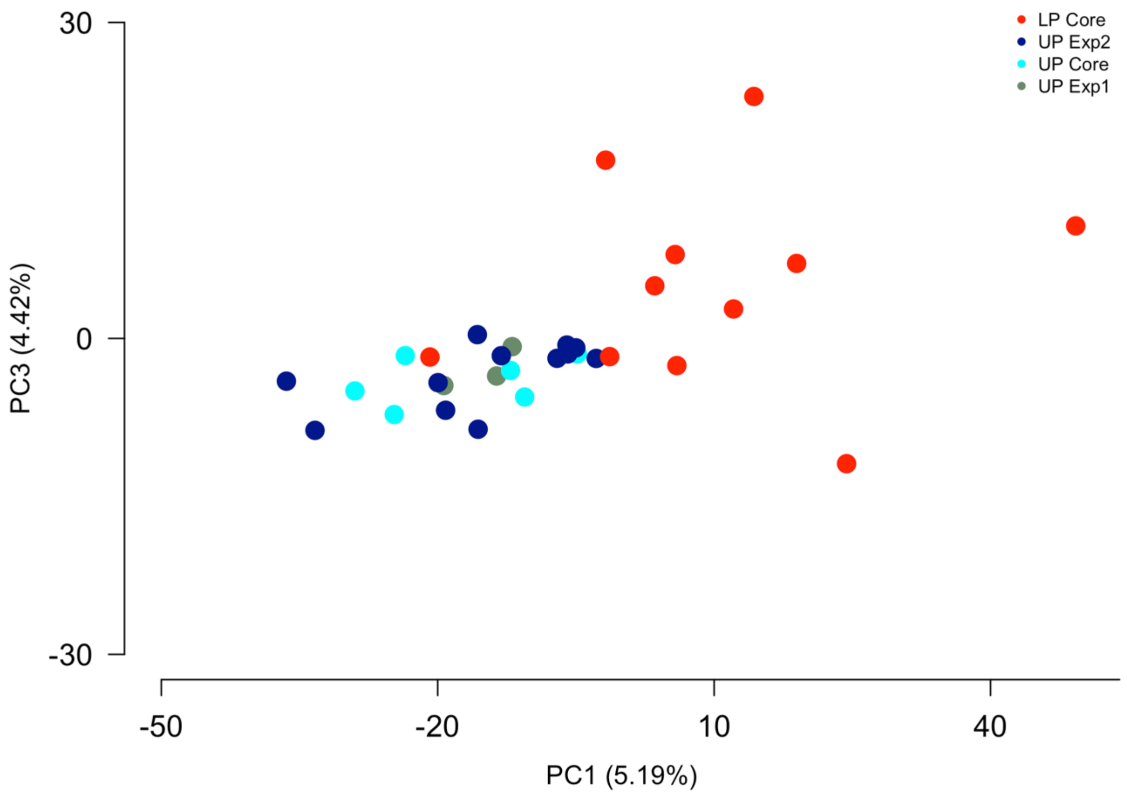

(f) PCA Outlier removed – PC2 and PC3

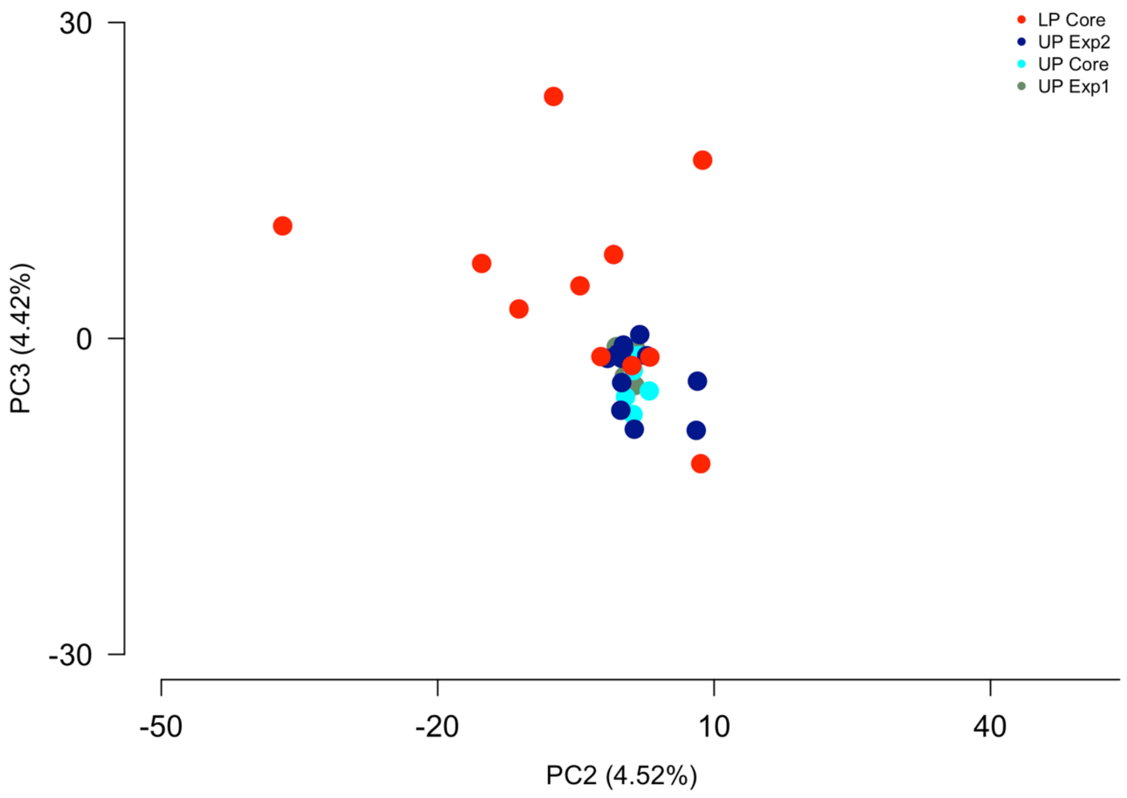

Table S2. Our principal component analysis (PCA) using PC1-PC3 (see main text for description and Figure S1) recovered an outlier individual in the lower peninsula (“LP”) historical (“Hist”) population. We therefore reanalyzed the data without this outlier individual (individual was MZ11379al from Table S1) and we recovered similar diversity statistics that we provided in the main text of the manuscript. Below we show the analysis for “All specimens” (including the outlier individual) and the reanalysis excluding this individual (“Outlier removed”), which yielded similar diversity statistics (shown below). We therefore retained this specimen in the final analysis that is shown in the main text of the manuscript (and below in “All specimens”). Sampled populations in northern Michigan shown here include UP Core (Menominee county), UP Exp1 (Schoolcraft county), UP Exp2 (Chippewa county), and LP Core (Cheboygan county). Summaries of genetic diversity (average observed heterozygosity (Hobs), average nucleotide diversity (Pi)) per sampled population are shown.

	**All specimens**				**Outlier removed**		
**Population**	**Pi**	**Var**	**StdErr**		**Pi**	**Var**	**StdErr**
LP Core	0.14551	0.01861	0.00152		0.14655	0.0189	0.00154
UP Exp2	0.11902	0.02466	0.00175		0.12102	0.02483	0.00177
UP Core	0.10699	0.03356	0.00204		0.10881	0.03393	0.00207
UP Exp1	0.09503	0.04623	0.00242		0.09666	0.04687	0.00246
	**Obs_Het**	**Var**	**StdErr**		**Obs_Het**	**Var**	**StdErr**
LP Core	0.129	0.01859	0.00152		0.13097	0.01931	0.00156
UP Exp2	0.11743	0.03217	0.002		0.11941	0.03247	0.00202
UP Core	0.10245	0.04021	0.00223		0.10418	0.04071	0.00227
UP Exp1	0.09401	0.05299	0.00259		0.09562	0.05374	0.00263
